# Engineering tissues with a perfusable vessel-like network using endothelialized alginate hydrogel fiber and spheroid-enclosing microcapsules

**DOI:** 10.1016/j.heliyon.2016.e00067

**Published:** 2016-02-02

**Authors:** Yang Liu, Shinji Sakai, Masahito Taya

**Affiliations:** Division of Chemical Engineering, Department of Materials Engineering Science, Graduate School of Engineering Science, Osaka University, 1-3 Machikaneyama-cho, Toyonaka, Osaka 560-8531, Japan

**Keywords:** Engineering, Biological sciences, Biomedical engineering, Biomaterials, Tissue engineering

## Abstract

Development of the technique for constructing an internal perfusable vascular network is a challenging issue in fabrication of dense three-dimensional tissues *in vitro*. Here, we report a method for realizing it. We assembled small tissue (about 200 μm in diameter)-enclosing hydrogel microcapsules and a single hydrogel fiber, both covered with human vascular endothelial cells in a collagen gel. The microcapsules and fiber were made from alginate and gelatin derivatives, and had cell adhesive surfaces. The endothelial cells on the hydrogel constructs sprouted and spontaneously formed a network connecting the hydrogel constructs with each other in the collagen gel. Perfusable vascular network-like structure formation after degrading the alginate-based hydrogel constructs by alginate lyase was confirmed by introducing solution containing tracer particles of about 3 μm in diameter into the lumen templated by the alginate hydrogel fiber. The introduced solution flowed into the spontaneously formed capillary branches and passed around the individual spherical tissues.

## 1 Introduction

The purpose of tissue engineering is the creation of three-dimensional (3D) tissue constructs that will benefit human health [1, 2]. To date, the tissue constructs of quite thin [3, 4, 5, 6] and/or those composed of the cells with a low oxygen demand [7, 8, 9, 10] have been successfully fabricated. The fabrication of dense tissues faces problems in developing methods for constructing perfusable vascular-like network inside them [11]. Among varieties of reported approaches for solving the problem, a feasible approach is based on the combination of following two approaches: 1) Development of tubular structure (several hundred micrometer to several millimeter in diameter) large enough to flow medium directly from medium reservoirs in hydrogels using appropriate templates [12, 13, 14, 15, 16, 17], and 2) spontaneous formation of capillary (less than dozens micrometers in diameter) network in hydrogels by the original ability of vascular endothelial cells [13, 14, 15, 17, 18, 19, 20]. It is expected that the network structure can be developed by combining the two approaches like a trunk and branches of a tree. In the preceding reports [14, 15, 17, 18, 20], the capillary branches formed randomly despite they were induced for transporting oxygen and nutrient to parenchymal cells. For more effective oxygenation and nutrition of the parenchymal cells, the capillary branches should be induced closer to the parenchymal cells.

**Fig. 1:**
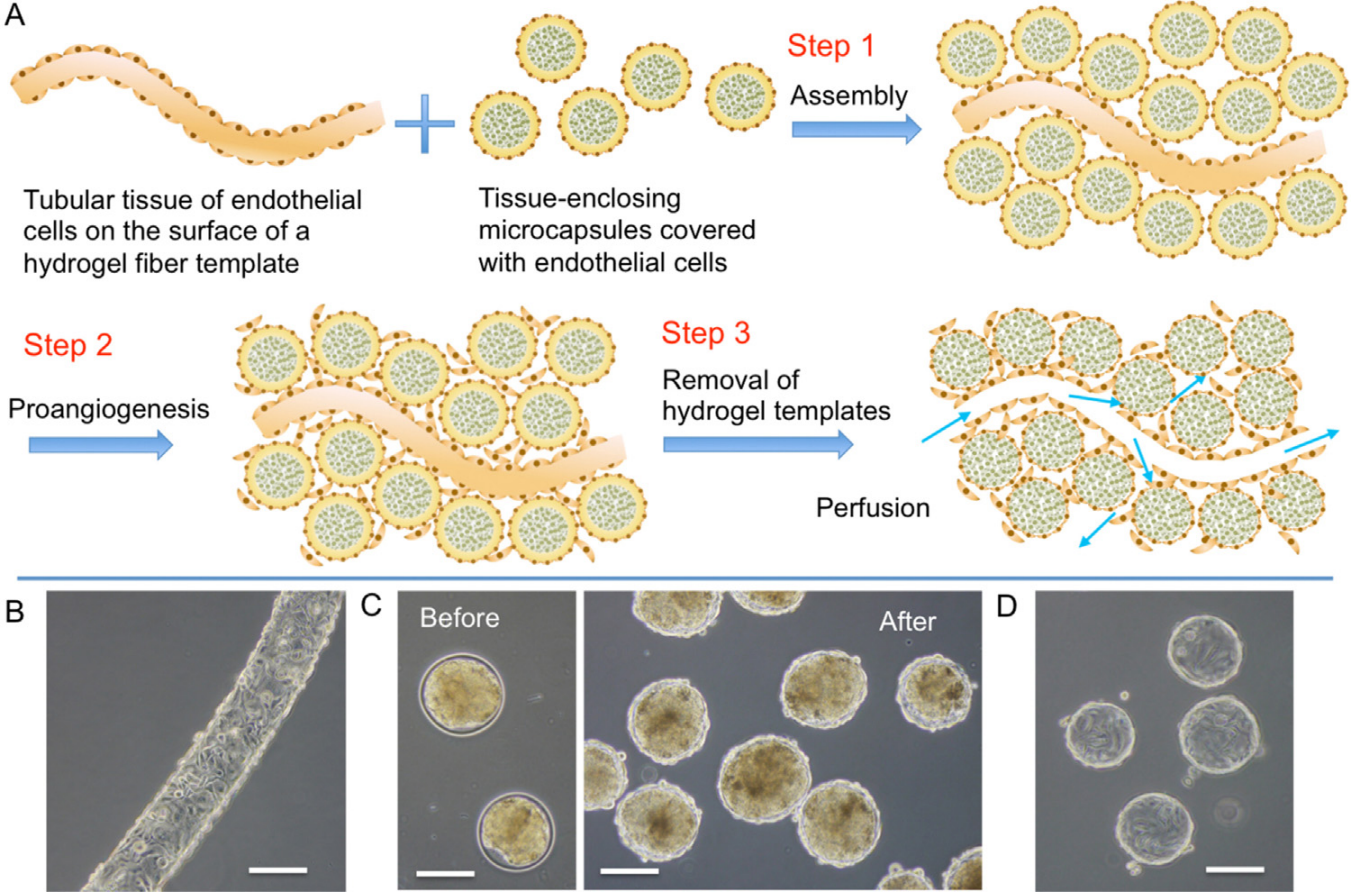
(A) Schematic of the assembly of hydrogel microcapsules with enclosed spherical tissues and a hydrogel fiber in collagen gel, both covered with endothelial cells in advance. Microphotographs of (B) a hydrogel fiber covered with HUVECs, (C) microcapsules enclosing spherical tissues of HepG2 cells at 6 days of culture before and after covering with HUVECs, and (D) empty microbeads covered with HUVECs. Bars: 200 μm.

The objective of the present study was to develop a method for fabricating a perfusable vascular network-like structure having a tubular construct and capillary branches close to spherical tissues of parenchymal cells. Our methodology for this objective is summarized in Fig. 1. First, we prepare hydrogel fiber and small-tissue enclosing microcapsules, both covered with vascular endothelial cells based on previously reported methods [21, 22]. The hydrogel constructs are prepared from alginate [23] and gelatin derivatives [24]. Then, they are assembled in a collagen gel (Step 1). After inducing sprouting and migration of the endothelial cells on the hydrogel constructs into the collagen gel resulting in the formation of capillary network between the hydrogel constructs in medium containing angiogenic factors (Step 2), the hydrogel templates, hydrogel fiber and microcapsules, are degraded using alginate lyase under a mild condition for cells (Step 3). As a result of the processes, we can obtain a perfusable vascular network-like structure having a perfusable tubular construct and capillary branches formed close to spherical tissues of parenchymal cells in the collagen gel.

Considering the potential application of the vascularized 3D tissue constructs, a variety of cells are possible candidates of the parenchymal and vascular cells. In this study, human liver carcinoma cell line HepG2 cells and human umbilical vein endothelial cells (HUVEC) were used as model cells for revealing the feasibility of our approach.

## 2 Materials and methods

### 2.1 Materials

Sodium alginate (MW: 70,000) was obtained from Kimica (Tokyo, Japan). Alginate lyase (from *Sphingobacterium multivorum*), gelatin (type A from porcine skin), and amylopectin (from maize) were purchased from Sigma-Aldrich (St Louis, MO, USA). Lecithin (from soybean), horseradish peroxidase (HRP), and H_2_O_2_ aqueous solution [30% (w/w)] were obtained from Wako Pure Chemical Industries (Osaka, Japan). Liquid paraffin and tyramine hydrochloride were purchased from Tokyo Chemical Industry (Tokyo, Japan). The derivatives of alginate, gelatin, and amylopectin possessing phenolic hydroxyl moieties (denoted as Alg-Ph, gelatin-Ph, and AP-Ph, respectively) were synthesized based on reported methods by the conjugation with tyramine hydrochloride using carbodiimide for synthesis of Alg-Ph [23] and gelatin-Ph [24], and carbonylimidazole for synthesis of AP-Ph [25]. The amounts of Ph moieties in Alg-Ph, gelatin-Ph, and AP-Ph were 1.5 × 10^−4^ mol Ph/g alginate, 9.4 × 10^−5^ mol Ph/g gelatin, and 3.6 × 10^−6^ mol Ph/g amylopectin, respectively.

### 2.2 Cell culture

HepG2 (Riken Cell Bank, Ibaraki, Japan) was cultured in Dulbecco's modified Eagle's medium (DMEM; Nissui, Tokyo, Japan) containing 10% fetal bovine serum (FBS; Gibco, Grand Island, NY, USA). Green fluorescent protein (GFP)-expressing human umbilical vein endothelial cells (GFP-HUVECs; Angio-Proteomie, Boston, MA, USA) were cultured in MCDB107 medium (Cell Science & Technology Institute, Miyagi, Japan) containing 10% FBS, 10 ng/mL human epidermal growth factor (Sigma-Aldrich), and 10 ng/mL human recombinant fibroblast growth factor-2 (ReproCELL, Kanagawa, Japan). The combination of angiogenic factors was based on a previous report [13], including vascular endothelial growth factor (VEGF; Gibco, Frederick, MD, USA), monocyte chemotactic protein-1 (MCP-1; Gibco), sphingosine-1-phosphate (S1P; Cayman Chemical, Ann Arbor, MI, USA), and phorbol 12-myristate 13-acetate (PMA; Abcam, Cambridge, MA, USA). VEGF, MCP-1, and PMA were used at 75 ng/mL, and S1P at 500 nM.

### 2.3 Empty microbead and tissue-enclosed microcapsule preparation

Empty microbeads and cell-enclosing microcapsules were prepared using an axisymmetric, flow-focusing droplet generation device designed in our laboratory [21]. Briefly, empty microbeads were obtained by extruding phosphate-buffered saline (PBS) containing 5% (w/v) gelatin-Ph and 100 units/mL HRP at 4.5 mL/h from a 26-G stainless steel needle into co-flowing immiscible liquid paraffin containing 4% (w/w) lecithin and H_2_O_2_. The emulsion system was centrifuged to collect the microbeads after mixing with PBS. To prepare tissue-enclosed microcapsules, first, cell-enclosing AP-Ph microbeads were prepared. A PBS solution containing 10% (w/v) AP-Ph, 100 units/mL HRP, and 4.0 × 10^7^ HepG2 cells/mL into co-flowing liquid paraffin containing lecithin and H_2_O_2_, and then processed as described for preparing empty microbeads. The diameter of the cell-enclosing AP-Ph microbeads was approximately 200 μm by adjusting the flow rate of the liquid paraffin mixture. Then, the cell-enclosing microbeads were mixed with PBS containing 1.5% (w/v) Alg-Ph, 0.5% (w/v) gelatin-Ph, and 100 units/mL HRP, and extruded into the same liquid paraffin mixture. The hydrogel membrane of the resultant microcapsules was no more than 20 μm in thickness. The microcapsules were incubated in medium for HepG2 cells growth. A hollow core in the microcapsules was established after the degradation of AP-Ph hydrogel template exposing in the medium containing FBS [25]. The viability of enclosed HepG2 cells right after the encapsulation was determined using trypan blue assay for the cells collected by degrading the microcapsules using alginate lyase. The growth profile of enclosed HepG2 cells was estimated based on the mitochondrial activity per microcapsule measured by a colorimetric method using a cell-counting kit-8 (Dojindo, Kumamoto, Japan) as previously described [21].

### 2.4 Hydrogel fiber preparation

Hydrogel fibers were prepared based on a reported method using a double co-axial cylinder system designed in our laboratory [15, 16, 22]. Briefly, PBS containing 4% (w/v) Alg-Ph and 100 units/mL HRP was extruded from a 25-G stainless steel needle at 0.15 mL/min into a co-flowing immiscible fluid of PBS containing 0.1% (w/v) gelatin-Ph and 0.3 mM H_2_O_2_ extruded from an 18-G stainless steel needle at 4 mL/min. The diameter of the resultant fibers was approximately 200 μm.

### 2.5 Covering with an endothelial cell layer

Empty microbeads, tissue-enclosing microcapsules or hydrogel fiber were respectively soaked in medium containing 8.0 × 10^5^ GFP-HUVECs/mL on non-adherent dishes for 3 h. For allowing homogeneous attachment of the GFP-HUVECs on the surfaces of the hydrogel constructs, the dishes were shaken slightly every 30 min. Then, the unattached cells were removed using a 100 μm cell strainer (BD Falcon, Durham, NC, USA).

### 2.6 Assembly of spherical vehicles and hydrogel fibers

Porcine skin collagen type I (Nitta Gelatin Inc., Osaka, Japan), 5 × DMEM, and neutralization solution were mixed on ice to produce a final collagen concentration of 2.1 mg/mL at pH 7.4. The empty microbeads or tissue-enclosed microcapsules with GFP-HUVECs were suspended in the collagen solution and poured onto a gelled thin collagen sheet formed in a well of 5 mm (length) × 5 mm (width) × 0.5 mm (depth). The hydrogel fiber covered with GFP-HUVECs was laid in the center of the well. After incubation for 15 min, the collagen gel was formed while containing the hydrogel fiber and microbeads or microcapsules. Then, additional collagen solution was poured onto the collagen gel to form a thin collagen gel layer on top. Subsequently, the resultant specimen was incubated in medium containing angiogenic factors at 37 °C in 5% CO_2_ and humidified air. The behaviors of GFP-HUVECs were observed under a fluorescence microscope (BIOREVE BZ-9000, Keyence, Osaka, Japan) and confocal-laser scanning microscope (CLSM; FluoView1000, Olympus, Tokyo, Japan).

The nuclei of HUVECs were stained with Hoechst 33342 (Dojindo, Kumamoto, Japan) for cell counting. Surface areas of the microbeads were calculated based on their diameter. The grow profile of HUVECs from the surface of microbeads were demonstrated by comparing the numbers of nuclei per unit of surface area of the microbeads in a same volume of the 3D assembly before and after 1 day of culture.

### 2.7 Immunohistochemical staining and hematoxylin and eosin staining

Immunohistochemical staining was performed on deparaffinized and rehydrated sections of 4-μm-thick. Endogenous peroxidase activity was quenched by incubation for 10 min in 3% H_2_O_2_. Antigen retrieval was performed by incubation for 10 min at 98 °C in 10 mM sodium citrate (pH 6.0). The sections were blocked in 5% goat serum (Sigma) for 1 h. Then, the sections were incubated with an anti-CD31 (89C2) mouse monoclonal antibody (1:100; Cell Signaling Technology, Danvers, MA, USA) overnight at 4 °C, followed by a peroxidase-conjugated secondary antibody (1:200; KPL, Gaithersburg, MD, USA), and visualized with a 3, 3′-diaminobenzidine (DAB) kit (Nichirei, Tokyo, Japan). Cell nuclei were stained with Mayer's hematoxylin solution (Wako) according to routine procedures. Hematoxylin and eosin staining was also performed according to routine procedures using the same paraffin-embedded sections mentioned above.

### 2.8 Fluorescent particle perfusion

**Fig. 2:**
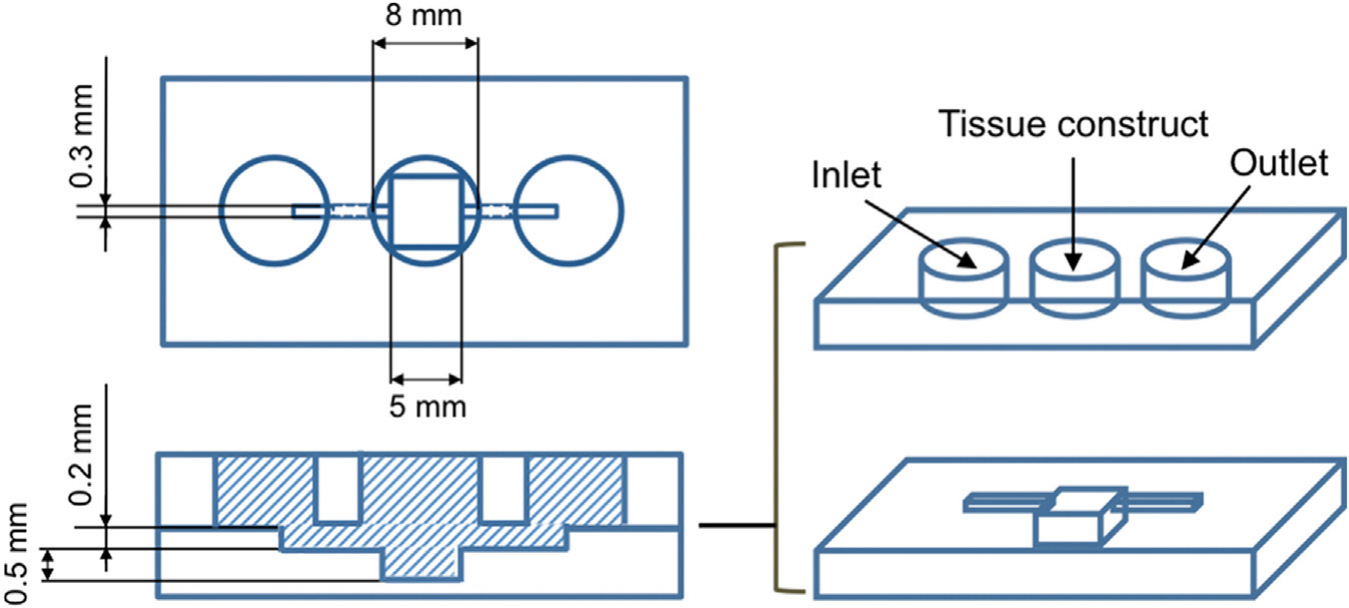
Schematic of the PDMS device used for fabrication of a dense tissue construct and perfusion experiment.

The specimen for this experiment was fabricated by assembling the tissue-enclosing microcapsules covered with GFP-HUVECs in a collagen gel at 50% (vol.) with a hydrogel fiber covered with GFP-HUVECs in a mold of 5 mm × 5 mm × 0.5 mm (height; Fig. 2). After the endothelial cell network formed among the microcapsules and the hydrogel fibers at 1 day of culture in medium containing angiogenic factors, hydrogel fiber and membrane of microcapsules were removed by using 0.025 mg/mL alginate lyase. Then, the tissue specimen was cultured for an additional day. Fluorescent microparticles of 3 μm in diameter (Polysciences, Warrington, PA, USA) were suspended in PBS and perfused into the cavity of a tubular tissue of endothelial cells formed after degrading the hydrogel fiber crossing the tissue constructs by introducing the solution from the inlet in the polydimethylsiloxane (PDMS) device (Fig. 2). The surface level of the solution was set to be 5 mm higher than the level of the cavity of the tubular tissue. The solution was drawn by gravity and kept flowing through the tubular cavity for several minutes.

## 3 Results and discussion

### 3.1 Vessel-like network formation among empty microbeads

**Fig. 3:**
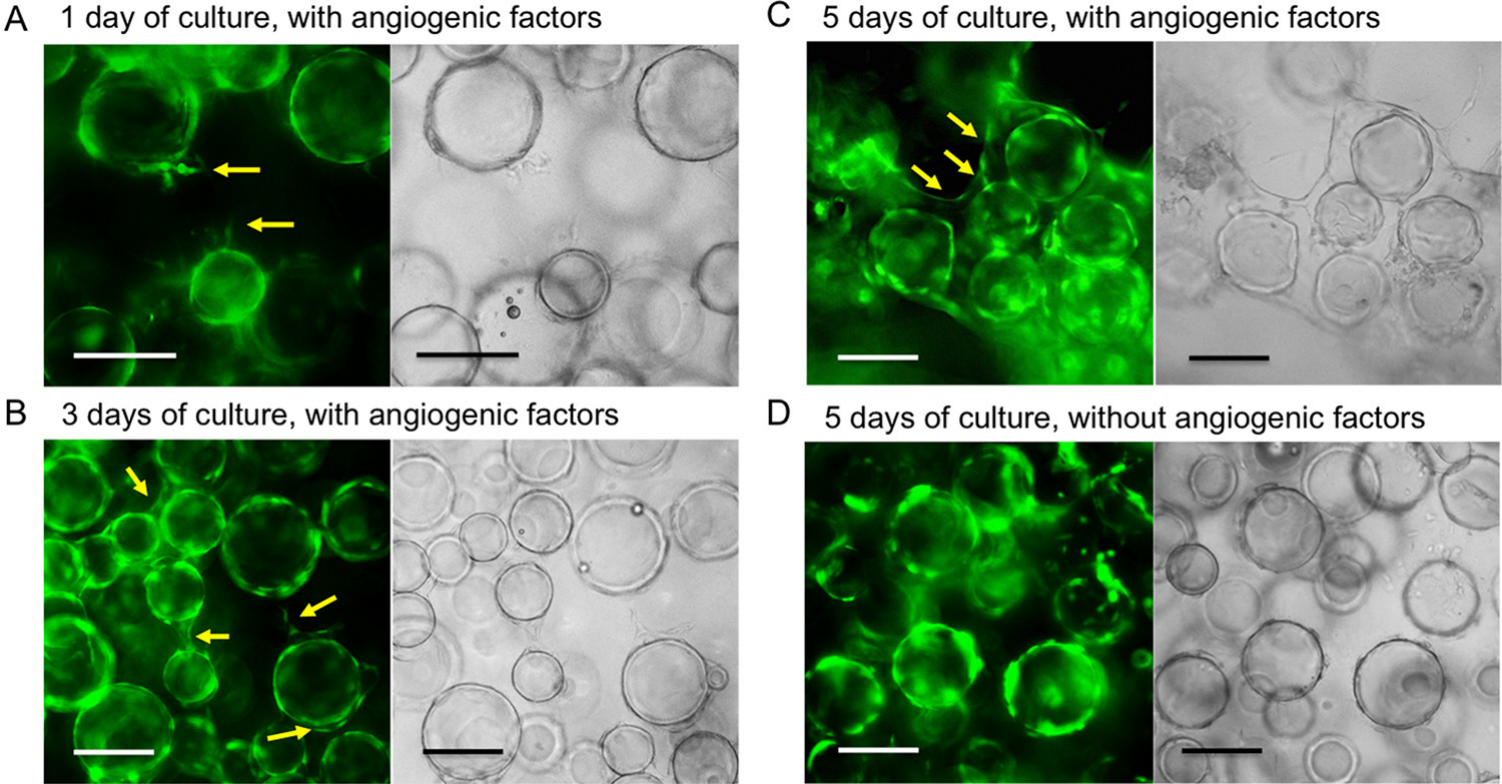
Fluorescence (left) and transmitted light (right) microphotographs of 33% (vol.) gelatin-Ph microbeads covered with GFP-HUVECs at (A) 1 day, (B) 3 days, and (C) 5 days of culture in the collagen gel using medium containing angiogenic factors, and at (D) 5 days of culture using medium without angiogenic factors. Arrows indicate the vascular structures formed spontaneously by the endothelial cells from the pre-existing GFP-HUVECs. Bars: 200 μm.

**Fig. 4:**
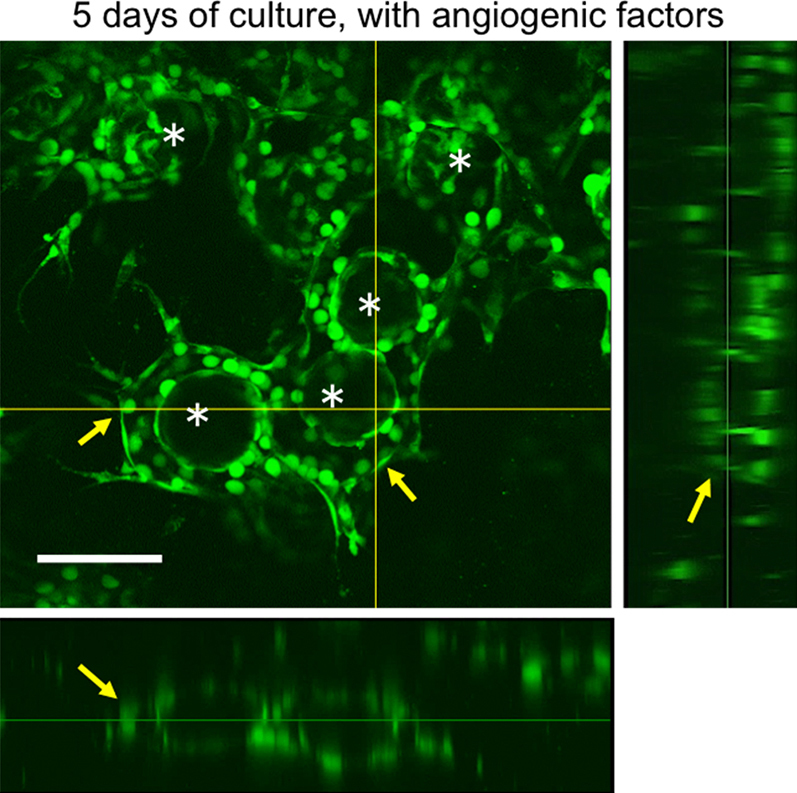
CLSM image of 33% (vol.) empty microbeads covered with GFP-HUVECs at 5 days of culture in the collagen gel using medium containing angiogenic factors. Arrows indicate lumen-like structures formed by GFP-HUVECs around the microbeads. Asterisks indicate the microbeads. Bars: 200 μm.

First, we examined the effect of the density of spherical vehicles covered with GFP-HUVECs in the collagen gel on the vessel-like network formation. In this experiment, we used empty gelatin microbeads covered with GFP-HUVECs. The study of using empty microbeads enabled to evaluate the behaviors of endothelial cells without any effects of co-existing parenchymal cells. From the results obtained at 33% and 67% contents of microbeads in total volume of specimen (expressed as 33% (vol.) and 67% (vol.), respectively), we found the effect of the contents on the time necessary for a formation of the vessel-like network. When the microbeads were embedded at 33% (vol.), sprouting of several endothelial cells into the surrounding collagen gel from the surfaces of the microbeads was observed at 1 day of culture in medium containing the angiogenic factors (Fig. 3A). Some endothelial cells from the adjacent microbeads were attached with each other at 3 days of culture (Fig. 3B). A neo-formed network of endothelial cells connected the majority of the microbeads at 5 days of culture (Fig. 3C). In contrast, poor cell migration was observed at 5 days of culture in medium without angiogenic factors (Fig. 3D). Similar phenomena caused by angiogenic factors have been reported in previous studies [13]. An interesting structure of the neo-formed, vessel-like network was revealed by imagine with a confocal-laser scanning microscope (CLSM; Fig. 4). An additional endothelial cell layer appeared around the microbeads in the ambient collagen gel. In fact, this cell layer and the cell layer on the surface of the microbeads were the two sides of an integral tubule of endothelial cells (Fig. 4, arrows). The majority of empty microbeads were surrounded by these new tubules. The tubules connected with each other to create a vessel-like network.

**Fig. 5:**
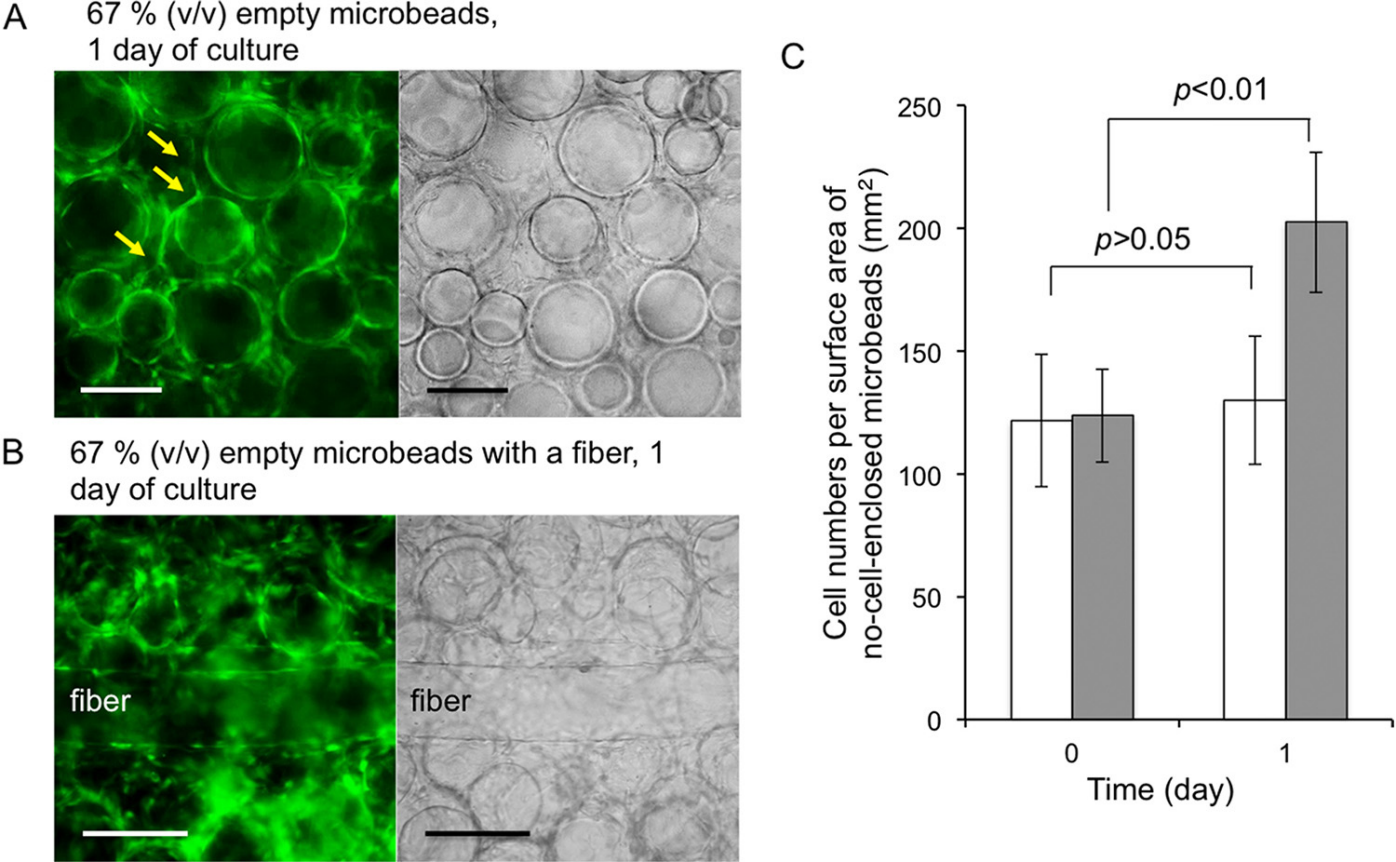
Fluorescence (left) and transmitted light (right) microphotographs of (A) 67% (vol.) empty microbeads covered with GFP-HUVECs at 1 day of culture in the collagen gel, (B) 67% (vol.) empty microbeads and a hydrogel fiber both with GFP-HUVEC layers at 1 day of culture in the collagen gel. Arrows indicate the branches of endothelial cells sprouted spontaneously from the pre-existing GFP-HUVEC layers. (A, B) Bars: 200 μm. (C) HUVEC growth profile from the surfaces of the microbeads that were embedded in the collagen gel at 33% (vol.) (white) and 67% (vol.). Error bars indicate the standard deviation.

The time necessary for the formation of the connecting network of endothelial cells decreased to 1 day with increasing the density of microbeads to 67% (vol.) (Fig. 5A). We further increased the density of the microbeads in expectation of decrease in the time necessary for the network formation. However, we could not get obvious results because of the difficulty in observation of individual microbeads using a microscope due to stacking of them. Focusing on the growth of endothelial cells on the surfaces of these microbeads embedded in collagen gel, obvious growth was not detected in the cells of 33% (vol.) microbeads in collagen gel after 1 day of culture. In contrast, the cells of 67% (vol.) microbeads in collagen gel increased to 1.6-fold (Fig. 5C). We believe that the promotion of vessel-like network formation is not simply caused by shortening the distance among each microbead in the higher density of microbeads covered with endothelial cells. One possible reason is the increase in the concentration of angiogenic factors secreted by the endothelial cells themselves. The enhancement of vascularization by autocrine factors of endothelial cells has already been reported in previous studies [26, 27, 28].

Endothelial cell tubules were also formed around the hydrogel fiber covered with endothelial cells embedded with the empty microbeads covered with endothelial cells at 67% (vol.) microbeads (Fig. 5B) at 1 day of culture in the medium containing angiogenic factors. In contract, the endothelial cell tubule was not formed when a single hydrogel fiber was embedded in the collagen gel at 1 day of culture under the same condition. This phenomenon could also be explained by the accumulation of autocrine factors secreted by the endothelial cells themselves.

In this study, HUVECs were used as a model of endothelial cells for confirming the feasibility of our approach of making a perfusable vessel-like network in engineered tissues. Considering the large heterogeneity of endothelial cells from different tissues and organs [29, 30, 31], it should be better to use the endothelial cells suitable for individual purposes to make the tissue constructs with specific functions.

### 3.2 Vessel-like network formation among tissue-enclosing microcapsules

**Fig. 6:**
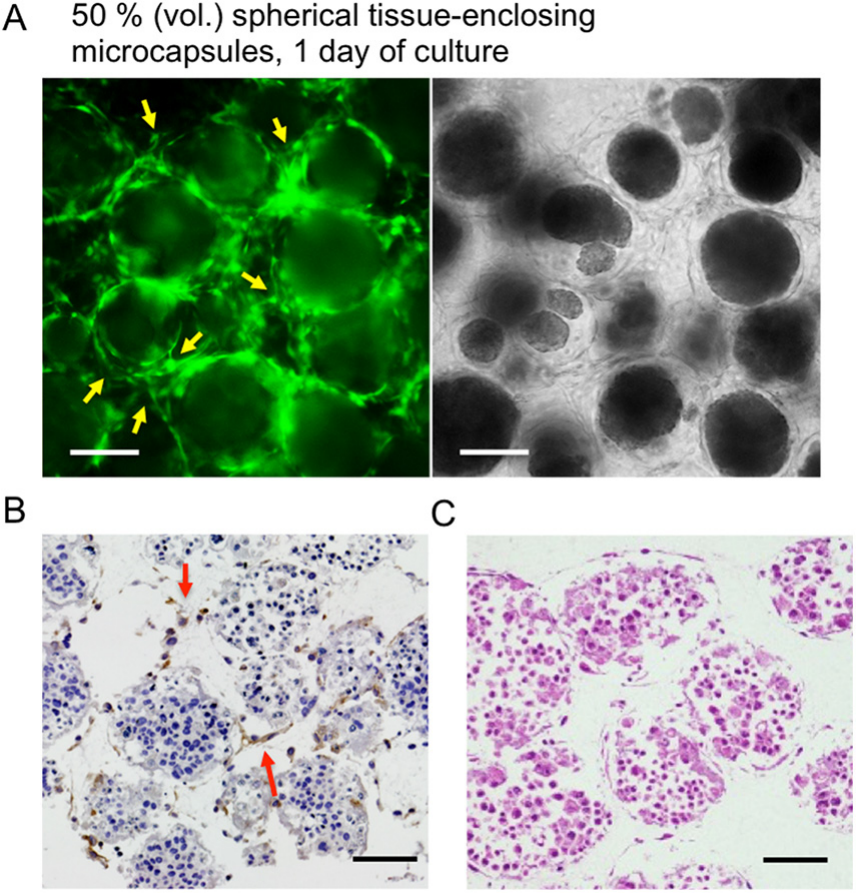
Fluorescence (left) and transmitted light (right) microphotographs of (A) 50% (vol.) microcapsules enclosing spherical tissues of HepG2 cells and covered with GFP-HUVECs at 1 day of culture in the collagen gel. Arrows indicate the branches of endothelial cells sprouted spontaneously from the pre-existing GFP-HUVECs. Bars: 200 μm. (B) Immunohistochemical staining of fabricated tissue construct. CD31-expressing HUVECs were visualized with DAB (brown). Cell nuclei were stained with Mayer's hematoxylin (purple). (C) Hematoxylin and eosin staining of fabricated tissue construct. (B, C) Bars: 100 μm.

Next, we assembled spherical tissue-enclosing microcapsules and a hydrogel fiber, both obtained from Alg-Ph and gelatin-Ph and covered with GFP-HUVECs, in a collagen gel. To make it easy to observe the vascular-like network, the density of the tissue-enclosing microcapsules was set to be 50% (vol.) of total volume. Before the assembly, the spherical tissues in the microcapsules were obtained by incubating the enclosed HepG2 cells (> 95% viability) for 6 days (Fig. 1B). The measurement based on the cellular mitochondrial activity per microcapsule indicated the enclosed HepG2 cells increased 3.3 times during 6 days of culture. It means the cell density in the microcapsule was around 1.3 × 10^8^ cells/mL. The discussion of suitable cell density for the spherical tissue is out of the scope of this paper. As expected, spontaneous network formation occurred at 1 day of culture in medium containing angiogenic factors. The majority of individual microcapsules were surrounded by endothelial cell tubules connecting between the microcapsules (Fig. 6A). This result demonstrated that the existence of tissue of HepG2 cells in microcapsules had no notable hindrance to the network formation, compared with the system using empty microbeads. The existence of vascular endothelial cells around individual spherical tissues and between neighboring spherical tissues was also confirmed by immunohistochemical staining for CD31 and hematoxylin-eosin staining in a vertical section of the specimen (Fig. 6B and C).

### 3.3 Perfusability study

**Fig. 7:**
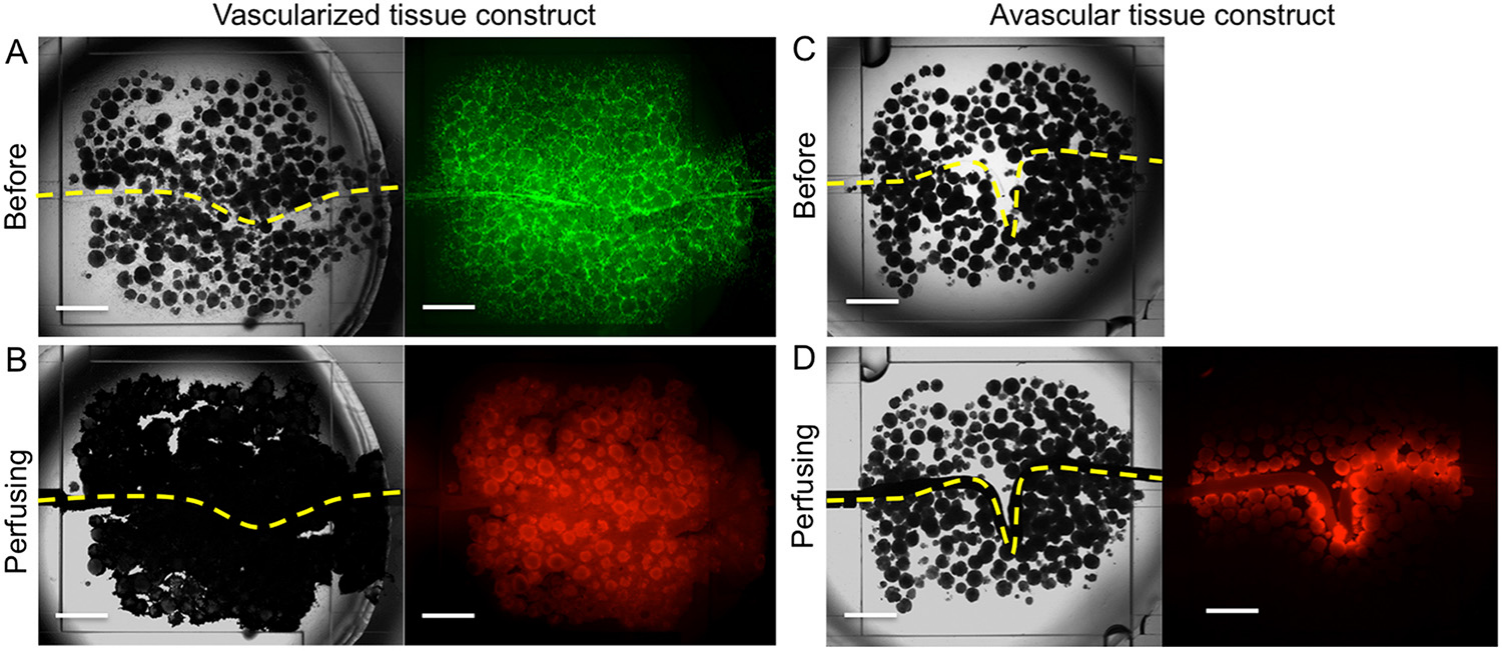
Transmitted light (left) and fluorescence (right) microphotographs of tissue constructs (A, B) with and (C, D) without the internal HUVEC (green in panel A) network (vascularized and avascular tissue constructs) (A, C) before and (B, D) during perfusion of 3 μm fluorescent microparticles (red in panels B and D). Dotted lines indicate the tubular cavity formed after degradation of hydrogel fibers. Bars: 1 mm.

**Fig. 8:**
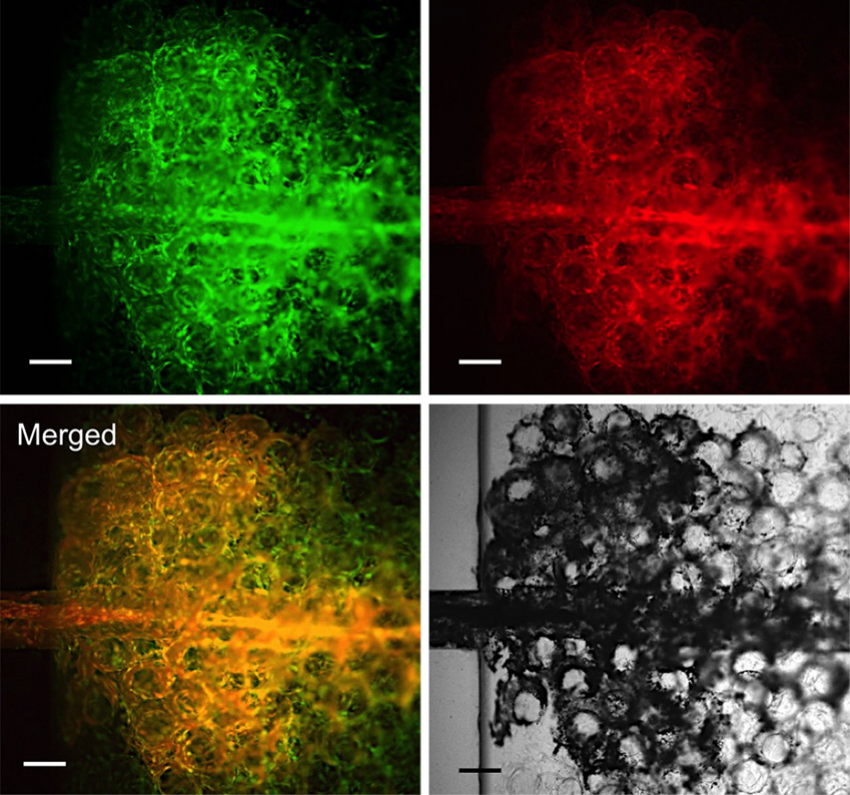
Fluorescence and transmitted light microphotographs during perfusion of the 3 μm fluorescent microparticles (red) into the tissue construct formed by assembly of empty microbeads made of gelatin-Ph and a hydrogel fiber made of Alg-Ph and gelatin-Ph, both covered with GFP-HUVECs (green). Bars: 200 μm.

Finally, we investigated the possibility of perfusion of solution in the spontaneously formed network of endothelial cells through the introduction of solution to the tubular cavity obtained by degrading the hydrogel fiber. A solution containing fluorescent microparticles of 3 μm in diameter was poured into the inlet well of the PDMS device for the introduction (Fig. 2). As shown in Fig. 7A and B, the fluorescent microparticles entered from the end of the tubular cavity flowed to the branched network of endothelial cells. The microparticles traveled around the individual spherical tissues through the network and finally flowed back to the tubular tissue (Supplementary Movie; Perfusion from the tubular tissue to capillary branches surrounding to spherical tissues). In contrast, the fluorescent microparticles only crossed the tubular cavity but could not reach the spherical tissues far away from the tubular cavity (Fig. 7C and D) in the specimen obtained by assembling the microcapsules and a hydrogel fiber, both non-covered with endothelial cells. These results demonstrate that the endothelial cells on the microcapsules and hydrogel fiber were essential for the formation of the perfusable vessel-like network between the spherical tissues. It may be thought that the degradation of the microcapsules by alginate lyase induced the cavity in which the solution flowed. However, it was denied by the result for the specimen obtained by assembling gelatin microbeads (non-degradable by alginate lyase) and the alginate-based hydrogel fiber both covered with GFP-HUVECs. The solution containing the fluorescent microparticles traveled around the individual gelatin microbeads when we introduced the solution into the tubular cavity obtained by degrading the hydrogel fiber using alginate lyase (Fig. 8).

In the current method, the hydrogel fiber covered with endothelial cells was used as a template for preparing the endothelialized tubular cavity into which solution was introduced from outer environment. Another reported method to prepare an endothelialized tubular cavity is an extraction of a gold rod covered with endothelial cells from collagen gel after transferring the cells to an ambient collagen gel [12]. An advantage of using the hydrogel fiber is the possibility of preparing curved tubular cavity due to the flexibility of the hydrogel fiber as shown in Fig. 7. It enables to increase the volume occupied by the single tubular cavity in fabricated tissue constructs with a single inlets and outlet. This feature allows fabrication of larger tissue constructs with a lower number of inlets and outlets, which is desirable for vascular anastomosis in transplantation of the fabricated tissue constructs. Considering the practical applications of tissue fabrication in transplantation, the tubular cavity templated by the hydrogel fiber of 200 μm in diameter is very small. The diameter of hydrogel fibers can be easily increased during the preparation process as reported previously [15, 16, 22]. Because of the feasibility demonstrated in the current study, our next objective will be fabrication of larger tissue constructs by increasing the number of hydrogel fibers and/or the diameter of the fiber to facilitate medium flow throughout the tissues.

## 4 Conclusions

In this study, we developed a method to fabricate a perfusable vessel-like network having a tubular construct and capillary branches in a dense tissue construct. We assembled microcapsules enclosing spherical tissues of HepG2 cells and a hydrogel fiber, both of which were covered with endothelial cells in the collagen gel. These endothelial cells spontaneously formed capillary branches, sprouted into the collagen gel and connected as a vessel-like network. Therefore, the individual spherical tissues requiring oxygenation and nutrition could be surrounded with capillary branches. A liquid carrier could be delivered and flowed through the network by introducing the solution into the tubular tissue obtained by degrading the hydrogel fiber template using alginate lyase. Considering the present results, we anticipate that the proposed method would greatly contribute to development of dense tissues *in vitro*.

## Declarations

### Author contribution statement

Yang Liu: Conceived and designed the experiments; Performed the experiments; Analyzed and interpreted the data; Contributed reagents, materials, analysis tools or data; Wrote the paper.

Shinji Sakai: Conceived and designed the experiments; Contributed reagents, materials, analysis tools or data; Wrote the paper.

Masahito Taya: Conceived and designed the experiments; Wrote the paper.

### Competing interest statement

The authors declare no conflict of interest.

### Funding statement

This work was supported by JSPS KAKENHI (grant A2511320) from the Ministry of Education, Culture, Sports, Science and Technology of Japan; and Kurata grant from the Kurata Memorial Hitachi Science and Technology Foundation. A part of this work was supported by "Nanotechnology Platform Project (Nanotechnology Open Facilities in Osaka University)" (No. F-14-OS-0019) from the Ministry of Education, Culture, Sports, Science and Technology, Japan.

### Additional information

No additional information is available for this paper.
