# Supplementary figures and images for: Engineering tissues with a perfusable vessel-like network using endothelialized alginate hydrogel fiber and spheroid-enclosing microcapsules
Source: Heliyon. 2016 Feb 2;2(2):e00067. doi: 10.1016/j.heliyon.2016.e00067 (PMC4946008; doi:10.1016/j.heliyon.2016.e00067)

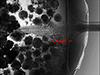

Supplement: Supplementary file 1 [file mmc1.jpg]
